# Assessment of global cardiac I-123 MIBG uptake and washout using volumetric quantification of SPECT acquisitions

**DOI:** 10.1007/s12350-012-9539-4

**Published:** 2012-06-06

**Authors:** Berlinda J. van der Veen, Imad Al Younis, Albert de Roos, Marcel P. M. Stokkel

**Affiliations:** 1 Department of Nuclear Medicine, Leiden University Medical Center, P.O. Box 9600, 2300 RC Leiden, The Netherlands; 2 Department of and Radiology, Leiden University Medical Centre, Leiden, The Netherlands; 3 Department of Nuclear Medicine, Netherlands Cancer Institute–Antoni van Leeuwenhoek Hospital, Amsterdam, The Netherlands

**Keywords:** I-123 *meta*-iodobenzylguanidine, myocardial innervation, SPECT, quantitative analysis

## Abstract

**Background:**

Assessment of cardiac innervation using single-photon emission computer tomography (SPECT) is less established than planar imaging, but may be more suitable for quantification. Therefore, a volumetric quantification of I-123 MIBG SPECT acquisitions was performed. Reproducibility, the effects of extra cardiac I-123 MIBG uptake and the relation with conventional planar indices were evaluated.

**Methods:**

54 patients referred for planar and SPECT I-123 MIBG acquisitions were included. Ellipsoidal or box-shaped volumes of interest were placed on the left ventricle, cardiac lumen, mediastinum, lung and liver. SPECT segmentation was performed twice in all patients. Indices were determined based on the heart-to-mediastinum (HM), myocardial wall-to-mediastinum and myocardial wall-to-lumen regions. HM ratios and washout rates were also determined based on anterior planar images.

**Results:**

Cardiac count densities were highly reproducible (CV 1.5-5.4, ICC 0.96-0.99) and inter-rater variability was low (CV 1.8-6.8, ICC 0.94-0.99). Mediastinal uptake was an important explanatory variable of uptake in the entire heart (early *R* ^2^ = 0.36; delayed *R* ^2^ =0.43) and myocardial wall (early *R* ^2^ = 0.28; delayed *R* ^2^ = 0.37). Lung washout was an explanatory variable of organ washout of the heart (heart *R* ^2^ = 0.38; myocardial wall *R* ^2^ = 0.33). In general, SPECT indices showed moderate-to-good correlations with the planar uptake (PCC 0.497-0.851).

**Conclusion:**

By applying a volumetric segmentation method we were able to segment the heart in all patients. SPECT I-123 MIBG quantification was found to be highly reproducible and had a moderate to good correlation with the planar indices.

## Introduction

Innervation imaging with radioiodinated *meta*-iodobenzylguanidine (I-123 MIBG) is the most widely used imaging technique to evaluate sympathetic nerve activity in cardiac diseases. The resemblance of MIBG to norepinephrine with respect to the molecular structure, synaptic uptake and intracellular storage makes it a suitable tracer to study sympathetic nervous activity.^1^-^4^

In general, the in-vivo visualization of cardiac innervation is evaluated on planar anterior images, which are acquired early and 3 to 5 hours after tracer injection. For quantification, myocardial and mediastinal regions of interest (ROIs) are drawn on anterior planar images, of which mediastinal ROI is thought to represent the non-specific I-123 MIBG uptake in soft tissue.^5^ Information on the distribution of neurons and function of the (re-)uptake-1 pathway is provided by the heart-to-mediastinum (HM) ratio, while the washout rate (WOR) provides information on the sympathetic drive. Both indices are commonly applied in I-123 MIBG imaging and the inter- and intraobserver variability of the HM ratio are considered low.^6^,^7^

Though planar imaging is widely used, quantification based on these acquisitions has important limitations. Precise quantification of myocardial counts is often complicated due to superimposition of adjacent organs onto the cardiac region. Furthermore, planar images do not contain three-dimensional information, making it difficult to assess regional innervation abnormalities.^8^,^9^ Single-photon emission computer tomography (SPECT) is known to overcome these problems. Still, the quantification of global cardiac innervation using SPECT is less established than planar I-123 MIBG imaging.

At present, SPECT imaging is considered informative in diseases like ischaemic heart disease, ventricular arrhythmias and diabetes mellitus were the cardiac innervation is affected in a heterogeneous manner.^9^ Consequently, this technique is increasingly used to assess the regional I-123 MIBG uptake.^6^,^8^,^10^-^13^ Using SPECT, it is possible to distinguish between myocardial wall, left ventricular lumen and surrounding organs. The ability to precisely localize these organs may suggest that SPECT acquisitions are also more suitable for the quantification of global innervation. To explore this hypothesis, we applied a simple volumetric quantification method on cardiac I-123 MIBG SPECT acquisitions. Global uptake in the heart, myocardial wall, ventricular lumen and surrounding organs were determined in clinical patient groups. Based on this data we studied reproducibility of SPECT segmentation, the effect of extra cardiac I-123 MIBG uptake and the relation between SPECT or the conventional planar indices.

## Materials and Methods

### Patient Population

The included population consisted of patients referred for I-123 MIBG acquisition prior to placement of a (biventricular) cardiac device (ICD) (group ICD, n = 28), to distinguish between neurodegenerative diseases (group NDD, n = 11), or after radiotherapy-chemotherapy (group RCT, n = 15). Since these groups all contain clinical patients, none of them can be considered a normal reference population. Still, the selection of these three populations ensures the inclusion of patients with a low cardiac I-123 MIBG uptake and a relatively normal I-123 MIBG uptake. All patients were referred for both planar and SPECT acquisitions in the period from November 2008 to March 2011 as part of their clinical work-up. Characteristics of the patient populations are provided in Table 1.

**Table 1:** Population characteristics

	ICD (n = 28)	NDD (n = 11)	RCT (n = 15)	*P* value^‡^
Age (years)	70 ± 6.1	64 ± 12.9	50 ± 10.0	<.001
Gender (% male)	20 (71%)	10 (91%)	0	<.001
Relevant medical history				
Autonomic dysfunction	0	9 (81%)	0	<.001
Hypokinesia/rigidity	0	5 (45%)	1 (7%)	<.001
Diabetes Mellitus	3 (11%)	2 (18%)	1 (7%)	.650
Hypertension	12 (43%)	0	2 (13%)	.010
Proven CAD	13 (46%)	1 (9%)	0	<.001
Infarction	17 (61%)	0	0	<.001
Heart failure	28 (100%)	0	0	<.001
Ventricular arrhythmia	18 (64%)	0	1 (7%)	<.001
(Biventricular) ICD	6 (21%)	0	0	<.001
Radio/chemotherapy	0	0	15 (100%)	<.001

^‡^Chi-square or ANOVA tests of the difference in distribution of various factors.

*CAD*, Coronary artery disease; *ICD*, implantable cardiac device.

### I-123 MIBG Acquisition Protocol

Patients were instructed to abstain from medication that could influence I-123 MIBG distribution (mainly antihypertensive drugs and tricyclic-antidepressants).^5^ Prior to I-123 MIBG administration patients underwent thyroid blocking by NaI droplets or KI capsules to prevent thyroid uptake of free radioiodine. An average dose of 185 MBq I-123 MIBG was slowly infused in 2 to 3 minutes. Anterior planar and SPECT images were obtained approximately 15 minutes (early) and 4 hours (delayed) after the injection. Anterior planar images were acquired using a dual-detector gamma camera (Toshiba, CGA 7200, Tokyo, Japan) equipped with a low energy high-resolution collimator (energy range up to 170 keV). Images were collected for 10 minutes with a 20% energy window centred on the 159 keV photo peak. The SPECT images were acquired with similar camera settings over 180° from right anterior oblique to left posterior oblique. Projections were made every 4° at 35 seconds per angle using a 128 × 128 matrix (zoom factor 1.5). SPECT data was reconstructed by ordered subset expectation maximization with 2 iterations and 10 subsets using a Gaussian post-processing filter (FWHM = 15 mm). No other filter or attenuation correction was applied.

### Planar Image Analysis

On the early and late images ROIs were defined by an experienced nuclear medicine technologist. The heart ROI was manually drawn to follow the external contours of the heart, thus including the myocardium and the left ventricular lumen. The apices of the lung and the mid-mediastinal line were used as anatomical landmarks for placement of a rectangular mediastinal ROI. The mean counts per pixel were determined in the early (*e*) and delayed (*d*) acquisitions for each ROI and corrected for decay. The early and delayed HM ratios were calculated as follows;

$$ {\text{HM}}_{\text{early}} = \frac{\text{eH}}{\text{eM}}\quad {\text{HM}}_{\text{delayed}} = \frac{\text{dH}}{\text{dM}} $$

According to the proposal for cardiac I-123 MIBG guidelines,^5^ WOR is defined as;

$$ {\text{WOR}} = \frac{{\left( {{\text{eH}} - {\text{eM}}} \right) - \left( {\frac{\text{dH}}{{0.5^{{^{{{\raise0.7ex\hbox{${\text{time}}$} \!\mathord{\left/ {\vphantom {{\text{time}} T}}\right.\kern-\nulldelimiterspace} \!\lower0.7ex\hbox{$T$}}}} }} }} - \frac{\text{dM}}{{0.5^{{^{{{\raise0.7ex\hbox{${\text{time}}$} \!\mathord{\left/ {\vphantom {{\text{time}} T}}\right.\kern-\nulldelimiterspace} \!\lower0.7ex\hbox{$T$}}}} }} }}} \right)}}{{\left( {{\text{eH}} - {\text{eM}}} \right)}} $$

With time indicating the difference between the early and delayed acquisition and *T* describing the physical decay of I-123. The factor 0.5^time/*T*^ in the denominator of the delayed part of this formula is used to account for the I-123 decay between the early and delayed acquisition.

### SPECT Image Analysis

SPECT image analysis was performed on a Syngo-MI workstation (Siemens Medical Solutions, USA). In a mid-ventricular short axis (SA) view an ellipsoid was placed to contain the entire heart. In the horizontal-long axis (HLA) and vertical-long axis (VLA) views the placement of this ellipsoid was evaluated and adjusted when needed to enclose the base and apex. A second ellipsoid was positioned to contain the left ventricular lumen. Based on this information, three-dimensional volumes of interest (VOIs) are automatically created for the heart and lumen. The activity in the myocardial wall was defined as ‘left ventricular counts minus lumen counts’. A box-shaped mediastinal VOI was placed conform the planar methodology. Uptake ratios and WOR were determined by applying the aforementioned formulas to three VOI combinations; HM, myocardial wall-to-mediastinum (MM) and myocardial wall-to-lumen (ML) regions. These values were compared to the planar indices to determine the discriminatory value of the SPECT indices in the patient populations.

The quantitative values determined on planar images are prone to count changes in the adjacent organs. To study the effects of I-123 MIBG uptake in adjacent organs in SPECT quantification, box-shaped VOIs were placed in centre of the liver and right lung. All box-shaped VOIs were between 10 and 15 cm^3^. For each VOI the size, uptake and washout were determined. The organ-specific washout was defined as;

$$ {\text{washout}} = \frac{{{\text{eOrgan}} - \frac{\text{dOrgan}}{{0.5^{{{\text{time/}}T}} }}}}{{{\text{eOrgan}} }} $$

**Figure 1:**
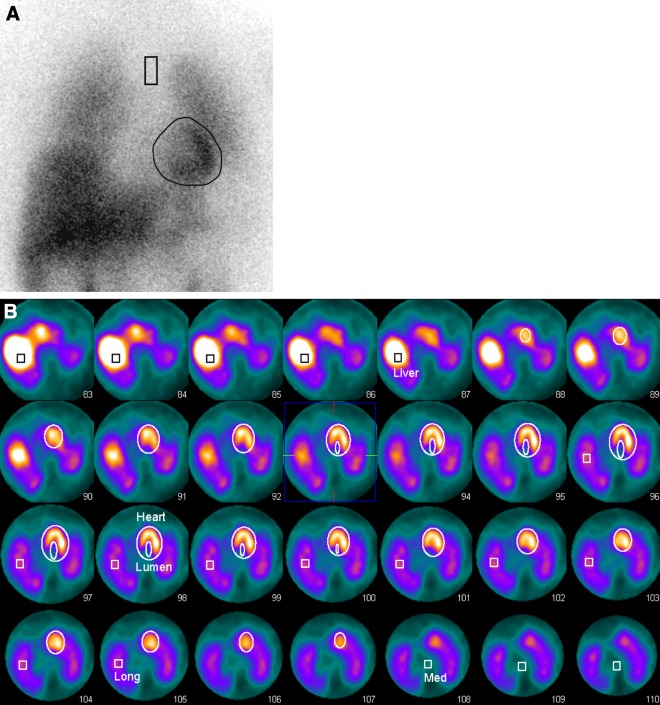
I-123 MIBG acquisition of a patient from the RCT population (age 64 years). This patient has breast cancer for which she receives chemo- and radiation therapy, and has no history of cardiac diseases. **A** The early planar image shows normal, intense uptake in the cardiac region (eHM = 1.94, dHM = 2.23, WOR = 0.02). **B** The VOIs for the left ventricle, lumen, mediastinum, right lung and liver are drawn into the SPECT acquisition. Based on these VOIs indices for the HM (eHM = 3.22, dHM = 2.97, WOR = 0.09), MM (eMM = 1.78, dMM = 1.51, WOR = 0.32) and ML (eML = 1.24, dML = 1.04, WOR = 0.82) are determined

**Figure 2:**
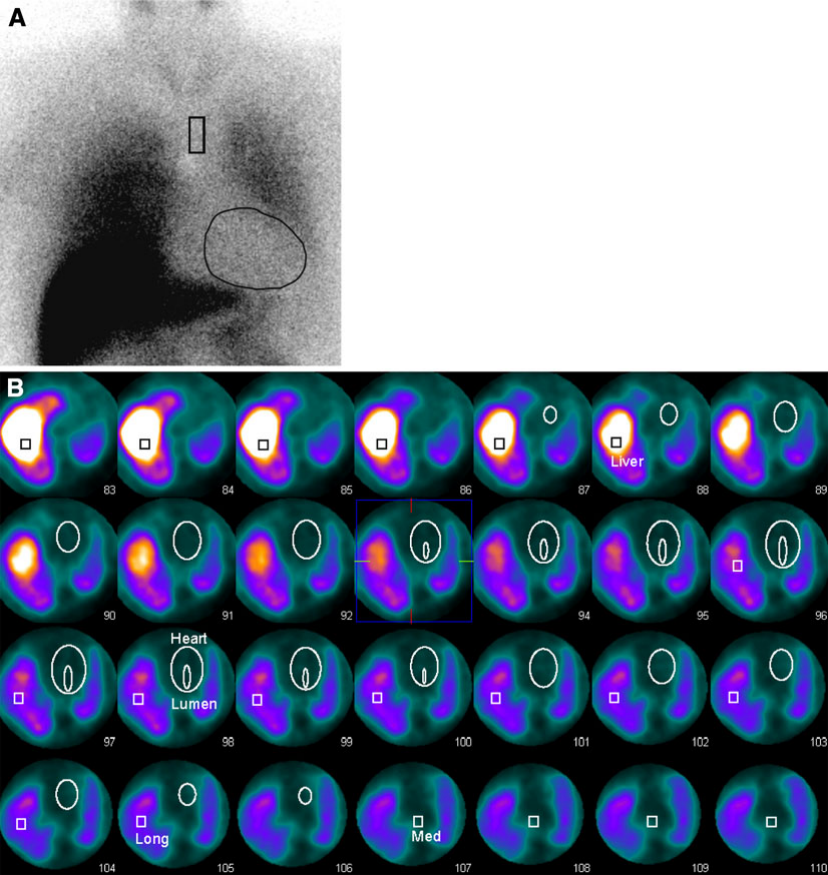
I-123 MIBG acquisition of a patient from the NDD population (age 60 years). This patient suffers from Parkinson’s disease and diabetes mellitus, but has no history of cardiac diseases. **A** The early planar image shows a severely reduced accumulation of I-123 MIBG in the cardiac region, but intense uptake in the liver and lung. (eHM = 1.21, dHM = 1.15, WOR = 0.42). **B** The VOIs for the left ventricle, lumen, mediastinum, right lung and liver are drawn into the SPECT acquisition. Based on these VOIs indices for the HM (eHM = 1.09, dHM = 0.91, WOR = 3.75), MM (eMM = 0.41, dMM = 0.26, WOR = −0.31) and ML (eML = 0.60, dML = 0.52, WOR = 0.08) are determined

Figure 1 provides an example of a normal planar and SPECT acquisition and Figure 2 provides an example of a patient with low cardiac count densities.

Reliability is an important aspect when manual segmentation is performed to assess SPECT data. Therefore, all acquisitions were segmented twice by the same experienced observer to evaluate the test-retest variability. The second segmentation was performed 2 weeks after the first segmentation to reduce observer bias. The manual SPECT-based segmentation was also performed by a second experienced observer to allow assessment of the inter-rater variability.

### Statistical Analysis

Statistical analyses were executed using the SPSS v16.0 software (SPSS inc. Chicago, USA). All continuous variables are expressed as ‘mean ± standard deviation’, and categorical data was expressed as frequencies and/or percentages. Continues variables were assessed for their distribution with the Shapiro-Wilk test.

Reliability can be described by a number of different statistical techniques. It is often considered a multi-factorial problem that is open to interpretation and is therefore assessed by multiple measures.^14^-^16^ In this study, test-retest and inter-rater reliability (i.e. reproducibility) were evaluated by the coefficient of variation (CV) and the intraclass correlation coefficient.

In addition to reproducibility of the segmentation factors like VOI size and I-123 MIBG uptake in surrounding organs may also influence quantification of counts within the heart. If a dependency exists between measured counts and either the size of the VOI or uptake in surrounding organs, the measurements are also considered less reliable. The relationship between VOI size and measured counts is determined by Pearson correlation coefficients (PCC). Stepwise multivariable regression analysis was used to determine the effects of I-123 MIBG uptake in the surrounding organs on the cardiac values. Regression analysis is described by the coefficient (*B*), standard error (se) and the total model fit (*R* ^2^). The global SPECT uptake ratios and WORs were compared to the widely used planar indices. Correlations between the planar and the SPECT values were expressed by Pearson correlation or Spearmann’s rank correlation, when appropriate.

## Results

### Reproducibility of VOI Size and Count Densities

In all acquisitions the segmentation was performed twice to assess the test-retest reliability. Size differences between the first and the second segmentations were found for all VOIs, as could be expected. The reproducibility of the box-shaped VOI size was considered relatively low (ICC ranging from −0.17 to 0.03 and CV ranging from 12.2% to 18.4%). In general, the elliptical cardiac VOIs (i.e. heart and lumen) had high ICC values indicating a good correlation, but also relatively high CV values suggesting high levels of variability between the segmentations. The size differences and the corresponding ICC or CV values are provided in Table 2a.

**Table 2:** Test-retest reproducibility of (a) VOI size, (b) count densities and (c) uptake ratios

	Mean	Difference^†^	CV	ICC	95% CI
*(a)*					
Early					
Liver	13.1 ± 1.3	2.52 ± 1.6	13.7	−0.14	−0.40–0.14
Lung	13.0 ± 1.3	2.32 ± 1.5	12.5	−0.09	−0.36–0.19
Mediastinum	12.7 ± 1.5	2.32 ± 1.7	18.4	0.00	−0.27–0.28
Heart	522.0 ± 237.5	77.2 ± 76.0	10.5	0.90	0.84–0.94
Lumen	10.4 ± 6.9	4.38 ± 4.7	29.8	0.65	0.46–0.78
Delayed					
Liver	13.2 ± 1.3	2.27 ± 1.7	12.2	−0.13	−0.39–0.15
Lung	13.3 ± 1.3	2.58 ± 1.8	13.7	−0.17	−0.43–0.11
Mediastinum	12.7 ± 1.5	2.39 ± 1.8	13.3	0.03	−0.25–0.29
Heart	519.0 ± 232.2	83.3 ± 107.1	11.5	0.84	0.75–0.91
Lumen	10.4 ± 6.9	4.03 ± 4.4	27.6	0.70	0.52–0.81
*(b)*					
Early					
Liver	28.0 ± 7.7	4.14 ± 3.6	10.4	0.79	0.63–0.87
Lung	12.8 ± 3.6	0.94 ± 0.9	5.2	0.93	0.88–0.96
Mediastinum	4.8 ± 1.3	1.25 ± 1.0	18.4	0.47	0.20–0.66
Heart	9.3 ± 3.6	0.20 ± 0.2	1.5	0.99	0.99–1.00
Lumen	5.0 ± 1.7	0.33 ± 0.3	4.7	0.96	0.93–0.98
Delayed					
Liver	26.6 ± 9.0	3.03 ± 2.5	8.1	0.91	0.81–0.95
Lung	10.5 ± 3.0	0.69 ± 0.6	4.6	0.95	0.91–0.97
Mediastinum	4.1 ± 1.0	0.50 ± 0.4	8.7	0.84	0.72–0.90
Heart	8.2 ± 4.4	0.23 ± 0.2	1.9	0.99	0.99–1.0
Lumen	4.4 ± 2.2	0.34 ± 0.3	5.4	0.97	0.95–0.99
*(c)*					
Early					
HM ratio	1.92 ± 0.4	0.48 ± 0.3	17.8	0.37	0.09–0.59
MM ratio	0.86 ± 0.3	0.25 ± 0.2	20.1	0.57	0.23–0.77
ML ratio	0.82 ± 0.2	0.13 ± 0.2	11.2	0.60	0.37–0.75
Delayed					
HM ratio	1.98 ± 0.7	0.26 ± 0.2	9.4	0.90	0.82–0.95
MM ratio	0.89 ± 0.4	0.15 ± 0.1	12.0	0.89	0.81–0.93
ML ratio	0.81 ± 0.2	0.14 ± 0.1	12.5	0.61	0.40–0.76

^†^One sample *T* test of the *absolute* differences between the two measurements, all differences were *P* < .001.

A low reproducibility of VOI size could result in poor reliability of the measured count densities. Nonetheless, the variability in count densities derived from the box-shaped VOIs was moderate, suggesting that the count densities obtained by the box-shaped VOI can be reliable. The cardiac VOIs provide highly reliable count densities in the test-retest evaluation (see Table 2b). The ICCs and CVs of the uptake ratios determined based on the three VOI combinations (HM, MM and ML) are provided in Table 2c. The inter-rater variability of the count densities for the cardiac VOIs was also considered low, as can be seen in Table 3. Both the CV and the ICC suggest a high degree of consistency in the values produced by the two raters. The variation among the uptake ratios is higher, as was also demonstrated in the test-rest analysis.

**Table 3:** Inter-rater reproducibility

	Mean	Difference^†^	CV	ICC	95% CI
Early					
Mediastinum	4.2 ± 0.9	0.73 ± 0.5	12.2	0.63	0.25–0.81
Heart	9.1 ± 3.5	0.35 ± 0.3	2.7	0.99	0.98–1.00
Lumen	4.8 ± 1.7	0.46 ± 0.3	6.7	0.94	0.89–0.97
HM ratio	2.17 ± 0.6	0.34 ± 0.3	11.0	0.80	0.57–0.90
MM ratio	1.01 ± 0.4	0.20 ± 0.2	14.0	0.77	0.58–0.87
ML ratio	0.87 ± 0.3	0.19 ± 0.1	14.9	0.52	0.29–0.69
Delayed					
Mediastinum	3.9 ± 1.0	0.42 ± 0.3	13.4	0.66	0.30–0.82
Heart	8.1 ± 4.3	0.21 ± 0.2	1.8	0.99	0.99–1.00
Lumen	4.4 ± 2.2	0.74 ± 0.6	6.8	0.97	0.94–0.98
HM ratio	2.08 ± 0.9	0.43 ± 0.5	14.6	0.75	0.52–0.87
MM ratio	0.95 ± 0.5	0.23 ± 0.3	17.2	0.74	0.56–0.84
ML ratio	0.84 ± 0.2	0.20 ± 0.2	17.3	0.51	0.28–0.69

^†^One sample *T* test of the *absolute* differences between the two measurements, all differences were *P* < 0.001.

When the total population is subdivided into the three patient groups, the test-retest ICC for the heart counts is 0.99 for each of the groups and the ICC for the ventricular counts VOI ranges from 0.90 to 0.98. The inter-rater ICC for the heart counts ranges from 0.97 to 0.99 and for the ventricular lumen from 0.88 to 0.99. So, both in the affected and near-normal populations SPECT segmentation of the heart can be considered reproducible.

### The Effect of VOI Size on Count Densities

The results of the test-retest analysis already suggest that there is no clear relationship between the VOI size and the count densities. Mean VOI sizes for all measurements are provided in Table 2a. When assessing the correlation between size and counts in all segmentations, no significant correlations were found in both early and delayed SPECT for liver (early PCC = 0.179, *P* = .066; delayed PCC = −0.015, *P* = .876), lung (early PCC = 0.131, *P* = .182; delayed PCC = 0.155, *P* = .112), heart (early PCC = −0.123, *P* = .209; delayed PCC = −0.143, *P* = .144) and mediastinal VOIs (early PCC = 0.030, *P* = .759; delayed PCC = −0.063, *P* = .524). Only for the lumen VOI moderate correlations were found in both early and delayed segmentations (early PCC = −0.344, *P* < .001; delayed PCC = −0.227, *P* = .020) suggesting a relationship between VOI size and measured counts.

### The Effect of Organ Uptake on Quantification

**Figure 3:**
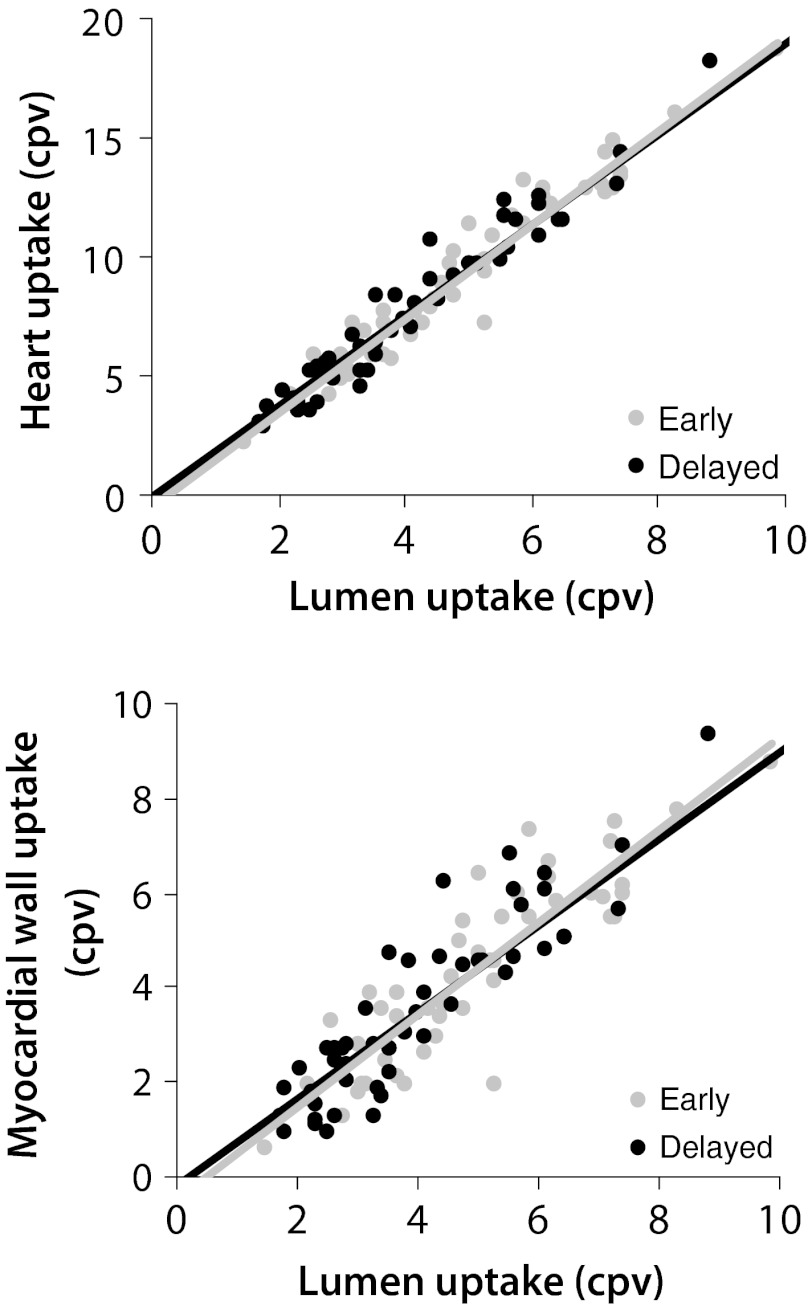
**A** The relationship between the heart uptake and the luminal count density on early (*R* ^2^ = 0.94) and delayed acquisition (*R* ^2^ = 0.96). **B** The relationship between myocardial uptake and the luminal count density on early (*R* ^2^ = 0.79) and delayed acquisition (*R* ^2^ = 0.85)

The effect of I-123 MIBG uptake in the surrounding organs was evaluated by multivariable regression analysis. The mediastinal uptake was an independent predictor of uptake within the entire heart (early *B* = 2.06, se = 0.38, *R* ^2^ = 0.36; delayed *B* = 2.74, se = 0.448, *R* ^2^ = 0.43) and in the myocardial wall (early *B* = 0.96, se = 0.22, *R* ^2^ = 0.28; delayed *B* = 1.27, se = 0.248, *R* ^2^ = 0.37). The lung washout was found to be an important explanatory variable of the organ washout in the entire heart (*B* = 0.58, se = 0.10, *R* ^2^ = 0.38) and the myocardial wall (*B* = 0.69, se = 0.14, *R* ^2^ = 0.33). For both cardiac uptake and washout, changes in liver uptake were not considered an important factor. The luminal count densities, although not included in the multivariate analysis, did show a strong relation to the uptake and washout in the heart or myocardial wall (Figure 3).

### Relationship Between Planar and SPECT Indices

**Figure 4:**
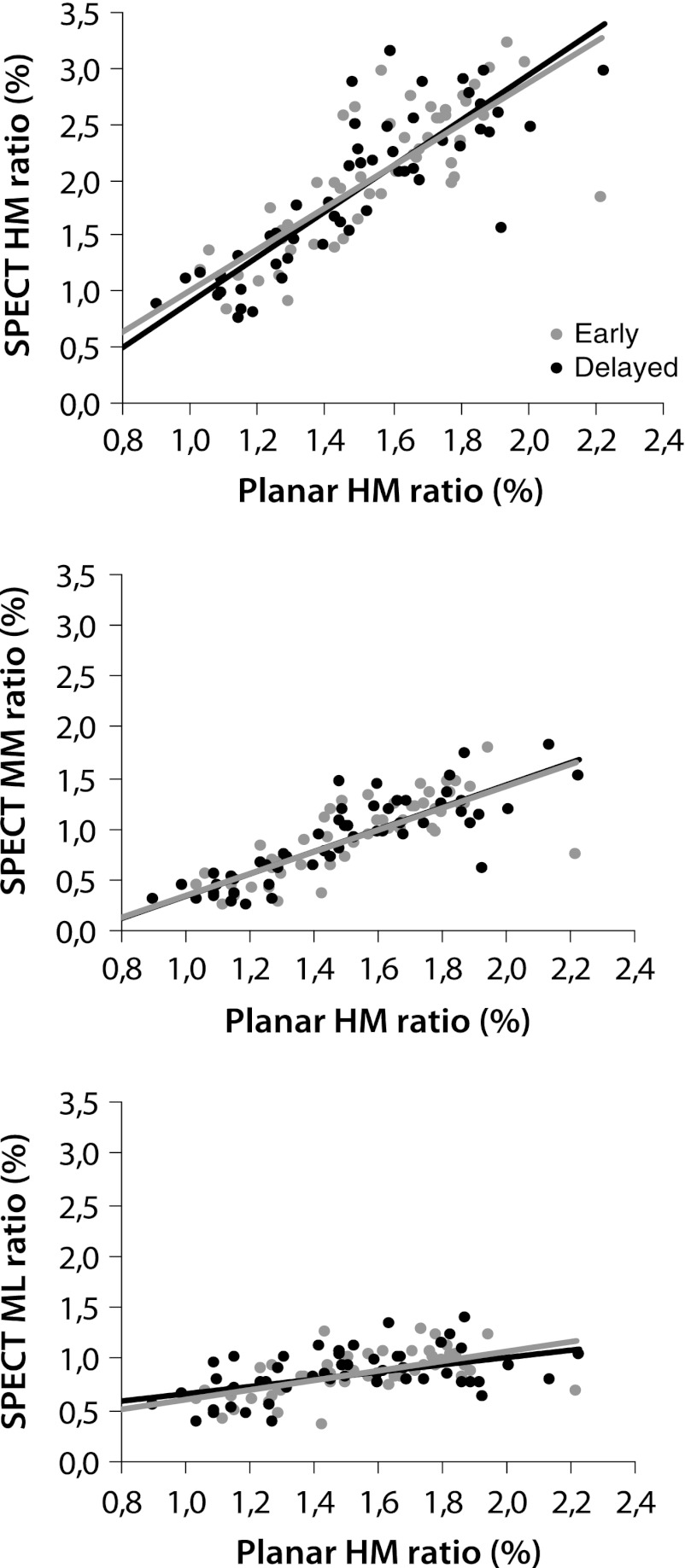
Relationships between the early and the delayed uptake ratios of the planar and the SPECT uptake indices determined by the four different methods. **A** HM (early *R* ^2^ = 0.64, delayed *R* ^2^ = 0.74). **B** MM (early *R* ^2^ = 0.60, delayed *R* ^2^ = 0.71). **C** ML (early *R* ^2^ = 0.33, delayed *R* ^2^ = 0.25)

The values for the planar and SPECT indices for the three different populations are provided in Table 4, as are the correlation between the SPECT and the planar indices. Especially the cardiac uptake ratios determined based on the SPECT data had a good correlation with the planar uptake values, as can be seen in Figure 4. The method using the ML ratio had the lowest regression coefficients, whereas the HM and MM had a moderate-to-good relationship with the planar indices. Using these latter two methods, it was also possible to discriminate between the three clinical patient populations (see Table 4).

**Table 4:** Relation between planar and SPECT indices

	Correlations^†^		Mean values for clinical populations^‡^			
	PCC^†^	*P* value	ICD	NDD	RCT	*P* value
Early						
Planar HM	–		1.53 ± 0.3	1.44 ± 0.3	1.71 ± 0.2	.024
SPECT HM	0.795	<.001	1.95 ± 0.5	1.70 ± 0.8	2.52 ± 0.3	.001
SPECT MM	0.771	<.001	0.91 ± 0.3	0.73 ± 0.5	1.20 ± 0.2	.006
SPECT ML	0.578	<.001	0.87 ± 0.2	0.69 ± 0.3	0.93 ± 0.2	.011
Delayed						
Planar HM	–		1.40 ± 0.2	1.38 ± 0.4	1.75 ± 0.2	<.001
SPECT HM	0.851	<.001	1.69 ± 0.6	1.63 ± 1.1	2.54 ± 0.3	<.001
SPECT MM	0.842	<.001	0.79 ± 0.3	0.71 ± 0.5	1.22 ± 0.2	.001
SPECT ML	0.497	<.001	0.85 ± 0.2	0.74 ± 0.3	0.92 ± 0.2	.139
Washout						
Planar HM	–		0.49 ± 0.38	0.48 ± 0.35	0.15 ± 0.13	<.001
SPECT HM	0.706	<.001	0.41 ± 0.65	0.97 ± 1.32	0.04 ± 0.32	.002
SPECT MM	0.359	.010	−0.71 ± 2.47	0.10 ± 0.56	1.38 ± 4.30	.044
SPECT ML	−0.145	.315	0.18 ± 2.06	−0.65 ± 1.90	1.56 ± 2.40	.163

*HM*, Heart-to-mediastinum ratio; *MM*, myocardial wall-to-mediastinum ratio; *ML*, myocardial wall-to-lumen ratio.

^†^Pearson correlation analysis or Spearman’s rank correlation between the planar and the SPECT for uptake and washout indices.

^‡^Anova or Kruskal-Wallis analysis to determine the difference between the mean values.

## Discussion

In this study, a simple volumetric-based segmentation of I-123 MIBG SPECT data was introduced to determine global uptake and washout in the entire heart and myocardial wall. Both the test-retest and the inter-rater reproducibility of the measured cardiac count densities were high, despite the low reproducibility of the VOI sizes. In both the affected and the near-normal populations SPECT segmentation of the heart can be performed and was found to be reproducible. The correlations between the widely used planar and SPECT indices was high for the uptake ratios determined based on the HM and MM regions. The differences between the three clinical patient populations were most profound when using the HM SPECT indices, which may suggest that this method has the best discriminatory value.

### Effects of Extra Cardiac I-123 MIBG Uptake

The extent to which external factors influence a measurement can be just as important for the clinical value of a technique as the reproducibility or the discriminatory value. Verberne et al.^17^ demonstrated that cardiac organ washout on planar images can be explained by a model containing mediastinum and lung washout. This implies that changes in myocardial count densities overtime are, to some extent, related to count changes in the surrounding organs.

Since cardiac segmentation can be performed more precisely on SPECT images due to the three-dimensional information content, it was anticipated that cardiac count densities determined on SPECT images were less prone to influences from surrounding organs. The results of this study suggest that mediastinal uptake was an important explanatory variable of cardiac uptake on both early and delayed images. Lung washout, on the other hand, was an independent predictor of the cardiac washout. Even though, the inclusion of mediastinal and lung counts into the cardiac VOI is limited when applying SPECT segmentation; these count densities do seem to correlate with the cardiac uptake and washout measures. Whether these effects are caused by technical problems in the segmentation, or reflect a true relation in the innervation processes within these organs remains unclear.

Liver uptake or washout had a smaller impact on quantification of cardiac counts, as was shown by both the study of Verberne et al^17^ and our study. Still, it is described that liver uptake has to be reckoned with when visually assessing I-123 MIBG SPECT images.^13^,^18^ Most often, uptake in the liver causes areas of reduced myocardial count densities, especially in inferior wall. In severe cases, the liver will superimpose on the myocardial wall making the entire inferior wall non-assessable.

### Lumen, Myocardial Wall or Entire Left Ventricle

In advance, it was hypothesized that SPECT imaging could also be more suitable for global quantification as it can distinguish between myocardial wall, ventricular lumen and surrounding organs. When using separate VOIs to describe the myocardial wall and lumen, it is possible to use the cardiac lumen as non-specific uptake measure which can be calibrated using a blood sample. This methodology was previously described by Somsen et al.^19^ Their study concluded that this is an accurate method to assess myocardial I-123 MIBG uptake.

The results of our study (Figure 3) demonstrate a strong positive relationship between the cardiac and the lumen count densities. This correlation is most likely the result of the spillover effect from the myocardial wall, and probably does not reflect a true relation between myocardial counts and the non-specific I-123 MIBG uptake. Additionally, the gathered experience suggests that lumen VOIs are more difficult to position than heart VOIs, especially in patients with small heart sizes or severely reduced cardiac uptake. These observations are supported by the reproducibility analysis, in which variability of lumen counts is higher than the variability of cardiac counts. Furthermore, a moderate correlation between the VOI size and the count densities was found for the lumen, suggesting that lumen counts are partly dependent on VOI size. All these findings suggest that lumen-based quantification of non-specific uptake may be less reliable, than for instance the mediastinal counts. Additionally, a recent study demonstrated in planar images that cardiac and mediastinal count densities are unrelated to changes in vascular I-123 MIBG activity, making a blood pool correction somewhat redundant.^20^

Uptake ratios as well as washout determined by the MM or HM regions showed a good relationship with the planar indices (Figure 4). The measurements based on the HM regions were considered to be more reproducible than the MM measures, and had a better discriminatory value. Still, more research has to be performed to establish the clinical value of both of these SPECT-based quantification methods.

### Other Methods to Assess Innervation Based on SPECT Data

The majority of the studies visually score cardiac uptake based on either the SA, HLA and VLA views or polarmap display.^6^,^8^,^10^-^13^,^22^-^24^ Automated evaluation or comparisons to a normal database are possible when polarmaps are used. All these methods rely on the basic principle that segmental uptake is scaled to the maximum count density within the heart. However, I-123 MIBG uptake can be abnormal throughout the myocardium creating a false representation of cardiac uptake. Additionally, false positive washout defects can be induced when discrepancies in delineation or orientation between the early and the delayed images are present. Thus, it is important to relate visual or automated scoring to count-based indices such as the HM ratio.^5^

At present, there are only a few studies that describe count-based indices derived from I-123 MIBG SPECT data. Druschky et al^21^ placed 33 small square ROIs in six SA slices to assess global and regional myocardial uptake. Results were expressed as percentage of the maximum ROI, and were used to calculate an inhomogeneity index. Since counts in each ROI are scaled to the maximum uptake, one expects that the scaling problem can arise in patients with overall reduced I-123 MIBG uptake. However, the idea of an inhomogeneity index is probably more suitable to described I-123 MIBG abnormalities in SPECT data, because there is no need to scale the data.

Somsen et al^19^ used single- and multi-slice SPECT data in which cardiac counts were related to either lumen or organ uptake. Their findings indicate that the single-slice SPECT method had a poor reproducibility, making it less suitable for global quantification. The multi-slice method, in which the entire cardiac volume is segmented, had a high reproducibility with CVs of <5%. The results found for the multi-slice method are comparable to the results found in our study.

### Clinical Application of I-123 MIBG SPECT

The regional assessment of cardiac innervation using SPECT is less established than planar I-123 MIBG imaging. In general, SPECT imaging is advisable in diseases were cardiac innervation is affected in a heterogeneous manner. There are, at present, a few clinical indications described for SPECT imaging which include ischaemic heart disease, ventricular arrhythmias and diabetes mellitus.^9^

The areas of sympathetic denervation often exceed beyond that of the perfusion defects, because sympathetic neurons are considered to be more sensitive to ischaemic events than cardiac myocytes. Furthermore, it is thought that chronic repetitive ischaemia may induce long-term sympathetic nerve dysfunction.^25^ These mechanisms also ensure that in the early stages of coronary artery disease or during frequent vasospastic events, sympathetic neurons can be affected without the presence of obvious perfusion defects.^11^,^22^,^26^ This heterogenic cardiac sympathetic innervation, in otherwise viable myocardium, is thought to be the source of electrical instability which is associated with the development of ventricular arrhythmias.^12^,^27^ The role of SPECT I-123 MIBG imaging in ischaemic heart disease and ventricular arrhythmias is therefore mainly focused on describing the regional sympathetic innervation status and detecting areas with perfusion/innervation mismatch.^10^,^28^ Cardiac autonomic neuropathy (CAN) in patients with diabetes mellitus, on the other hand, is associated with regional hyperactivity of the sympathetic nervous system, resulting in an electrical instability.^8^,^29^ It is recognized that planar I-123 MIBG imaging is useful to detect CAN and provide prognostic information on future cardiac events.^30^-^32^ However, planar indices are less sensitive in detecting small regional abnormalities which are often associated with diabetic neuropathy. Therefore, it was suggested by Hattori et al^8^ that I-123 MIBG SPECT imaging should be used to detect CAN. Still, the additive role of SPECT imaging in diabetes needs to be established.

### Study Limitations

In this study, a straightforward volumetric technique was introduced that is able to provide reproducible global I-123 MIBG parameters. The initial results indicate that it is possible to distinguish between different patient populations using these SPECT indices, and that these indices relate to the planar measures. Still, this study does not provide a validation of this quantitative method. Such a validation will require the comparison of the new SPECT-based method to a golden standard. At present, there are no measures that can serve as a true golden standard, only the prognosis of the patient can be used as such. The only other imaging technique that is able to directly assess cardiac sympathetic innervation is C-11 *meta*-hydroxyephedrine (HED) positron emission tomography (PET).^33^ This technique is, however, only available in a limited number of centres and is not yet considered to be standard clinical practice. Accordingly, more research has to be performed to establish the value of I-123 MIBG SPECT imaging over planar imaging in specific patient populations.

## Conclusion

By applying a simple volumetric segmentation method we were able determine global uptake and washout in the entire heart or myocardial wall in all patients. In general I-123 MIBG SPECT quantification was found to be highly reproducible, reliable and had a moderate-to-good correlation with the planar indices. Still, the additive value of I-123 MIBG SPECT quantification over planar imaging has to be established in specific patient populations.
